# GABA predicts visual intelligence

**DOI:** 10.1016/j.neulet.2016.07.053

**Published:** 2016-10-06

**Authors:** Emily Cook, Stephen T. Hammett, Jonas Larsson

**Affiliations:** Department of Psychology and CUBIC, Royal Holloway, University of London, Egham, TW20 0EX, United Kingdom

**Keywords:** GABA, Intelligence, Visual cortex, Surround suppression, Inhibition

## Abstract

Early psychological researchers proposed a link between intelligence and low-level perceptual performance. It was recently suggested that this link is driven by individual variations in the ability to suppress irrelevant information, evidenced by the observation of strong correlations between perceptual surround suppression and cognitive performance. However, the neural mechanisms underlying such a link remain unclear. A candidate mechanism is neural inhibition by gamma-aminobutyric acid (GABA), but direct experimental support for GABA-mediated inhibition underlying suppression is inconsistent. Here we report evidence consistent with a global suppressive mechanism involving GABA underlying the link between sensory performance and intelligence. We measured visual cortical GABA concentration, visuo-spatial intelligence and visual surround suppression in a group of healthy adults. Levels of GABA were strongly predictive of both intelligence and surround suppression, with higher levels of intelligence associated with higher levels of GABA and stronger surround suppression. These results indicate that GABA-mediated neural inhibition may be a key factor determining cognitive performance and suggests a physiological mechanism linking surround suppression and intelligence.

## Highlights

• Cortical GABA levels correlate with visuo-spatial IQ and surround suppression.
• Higher levels predict higher intelligence and stronger surround suppression.
• Results provide mechanism linking surround suppression and intelligence.
• Results suggest role of cortical GABA levels in determining cognitive performance.

## 1 Introduction

It has long been argued that intelligence represents some fundamental property of the brain [1], [2] that should also be reflected in low-level visual abilities. Links between sensory measures and intelligence have been documented in multiple domains [3], [4] and low level visual abilities and intelligence correlate in both typical individuals [5] and patient groups [6]. Recently, it has been suggested that the key factor linking intelligence and sensory performance is the ability to suppress irrelevant information, as evidenced by the correlations between intelligence and sensory suppression [7], [8]. However, the neural substrate of this link remains unclear. Visual surround suppression has been shown to correlate with differences in cortical GABA levels in individuals with schizophrenia relative to controls [9], suggesting a role for neural inhibitory mechanisms in mediating this link. Additionally, both GABA [10] and some measures of surround suppression [11] have been shown to decline with age, and GABA has been linked to age-related cognitive decline [12]. However, although individual variations in GABA have been shown to correlate with some perceptual abilities [13], [14], [15], other studies have failed to find a direct link between surround suppression and pharmacological interventions aimed at manipulating GABA levels [16], [17]. In this study, we measured individual variations in cortical GABA levels, visuo-spatial intelligence and perceptual surround suppression in a group of typical individuals, providing evidence that GABA-mediated neural inhibition is strongly linked to both visual intelligence and susceptibility to surround suppression.

## 2 Material and methods

### 2.1 Subjects

9 subjects (2 males) aged between 22 and 34 took part in this experiment. Subjects had normal or corrected to normal vision. Subjects were post-graduate students or post-doctorate researchers at the Psychology department of Royal Holloway, University of London. All subjects except one (one of the authors) were naïve to the purpose of the experiment.

### 2.2 Measurement of visual intelligence

The Matrix Reasoning subtest of the Wechsler Abbreviated Scale of Intelligence (WASI) was administered in accordance with the standardised procedures outlined in the WASI user’s manual [18]. This test measures visuo-spatial problem solving. All psychometric testing occurred in a single session. Psychometric scores were analysed as raw values, corresponding to the total number of correct verbal responses. The highest possible WASI score is 35.

### 2.3 GABA estimation

Estimates of GABA concentration in the visual cortex were obtained using the methods of Edden et al. [19]. Magnetic resonance spectroscopy (MRS) was performed using a 3T whole-body MR scanner (Magentrom Trio: Siemens, Erlagen, Germany) at Royal Holloway. Data collection for each participant took approximately 35 min and was completed in a single session. During collection of this data participants were instructed to lie as still as possible, no task was performed.

A T2 anatomical scan was performed to collect images in 3 planes for MRS voxel placement (in-plane resolution 0.4 × 0.4 mm, slice thickness 3 mm). Following visual inspection of this anatomical image the MRS voxel was placed over the primary visual cortex, identified by the calcarine sulcus. The voxel was placed so as to exclude as much cerebrospinal fluid as possible and care was taken to ensure the voxel did not extend into the dura.

Acquisition of MRS data was performed using the MEGA-PRESS sequence [20]. The following parameters were used; voxel size = 30 × 35 × 25 mm, repetition time (TR) = 2000 ms, echo time (TE) = 68 ms. MRS acquisition took approximately 15 min. An editing pulse was applied to the GABA signal at 1.9 ppm to isolate GABA signals from the spectra. A reference scan was also collected, for which no editing pulse was applied and water was unsuppressed. In total, 2 GABA scans and 2 reference scans were collected. GABA scans and reference scans were collected in alternating order, with a GABA scan always collected first.

MRS data was processed using the Gannet toolbox [19]. This analysis technique exploits the difference between the reference scan and the scan in which the editing pulse was applied to estimate the GABA signal separate from the underlying creatine signal at 3 ppm [20]. The final estimate of GABA concentration represents the peak of the GABA spectrum. The toolbox provides two estimates of GABA, one relative to water (GABA/H_2_0) and one relative to creatine (GABA+/Cr). GABA/H_2_0 was used due to the superior signal to noise ratio [21]. 2 estimates of visual cortex GABA concentration were obtained for each subject by comparing each of the 2 GABA scans with the reference scan immediately following it. Consistency across the 2 estimates indicated high reliability. A single GABA estimate from each subject was obtained by taking the average of these 2 estimates.

### 2.4 Measurement of perceptual surround suppression

Visual stimuli were displayed on an EIZO 660-M monochrome CRT monitor at a viewing distance of 57 cm. A chin rest was used to ensure head stabilization. Presentation of stimuli and acquisition of responses was carried out using Matlab 7.4.0 (R2007a) and MGL (http://www.pc.rhul.ac.uk/staff/J.Larsson/softare.html) run on a Linux operating system.

**Fig. 1:**
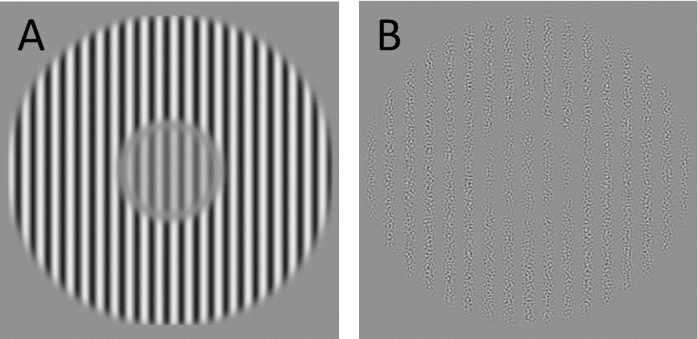
A. First order surround stimulus. Luminance defined sinusoidal grating. B. Second order surround stimulus. Contrast modulated sinusoidal grating.

The magnitude of perceptual surround suppression was measured separately for two types of visual stimulus patterns: luminance-defined (first-order; Fig. 1A) gratings and contrast-modulated (second-order; Fig. 1B) gratings, both of which are known to induce surround suppression [22], [23], [24]. Subjects performed a temporal two-alternative forced choice (2AFC) contrast matching task, judging which of two sequentially presented target stimuli had higher contrast. Surround suppression strength was measured by comparing contrast matching thresholds from a surround condition with those of a control condition with no surround. The 2 psychophysical measures were obtained in separate testing sessions, with first-order surround suppression measured in the first set of sessions. In total, each subject attended 8 60 min testing sessions.

Each task was carried out at a range of eccentricities (0°, 3°, 6°, 9°), in 2 quadrants of the visual field (upper left and upper right). Within each block of trials (60 trials), the stimuli were always shown at a single location. Within each surround suppression testing session, the stimuli were always of the same experimental condition (control or surrounded). Throughout each trial, a white 1° fixation cross was displayed at the centre of the screen. The background was a uniform gray with a luminance of 26.1 cd/m^2^.

First-order grating stimuli consisted of luminance-defined sinusoidal gratings (spatial frequency 2 cycles per degree) within a 3° wide circular aperture. Second-order grating stimuli consisted of contrast-modulated sinusoidal gratings generated as described in Larsson, Landy and Heeger [25] by modulating the luminance contrast of an isotropic band-pass filtered noise carrier (50% root mean square contrast, spatial frequency 8 cycle per degree, and bandwidth 1 octave). Modulator frequency was 2 cycles per degree and modulation contrast of the reference stimulus gratings was 90%. In the surround condition, the first target stimulus in each trial was surrounded by a high-contrast grating (first-order: 80% luminance contrast, second-order: 100% modulation contrast) with the same spatial frequency and phase as the target, displayed within an annulus (inner diameter 4°, outer diameter 11°). The target and surround gratings were separated by a 0.5° wide blank space with the same luminance as the background. In the control condition, both targets were shown without a surround. The edges of target and surround apertures were blurred to yield a soft edge. A 1° wide fixation cross was shown throughout each trial. On each trial, the first (reference) target stimulus was displayed for 0.5 s followed by an inter-stimulus interval of 0.5 s, after which the second (matching) target stimulus was shown for 0.5 s. Subjects indicated by pressing one of two buttons within 2.5 of stimulus onset whether the matching stimulus was higher or lower contrast than the reference stimulus. The contrast of the reference stimulus was fixed at 40%, while the contrast of the matching stimulus was adjusted using a 1-up, 1-down staircase. 5 blocks of 60 trials each were run for each stimulus eccentricity and quadrants. For each block, the contrast threshold was computed as the average matching stimulus contrast of the last 20 trials.

An index of first-order surround suppression magnitude *S1₁* was calculated by subtracting the ratio of the matching contrasts (averaged across blocks and hemifields) of the surround and control conditions from 1 [22].

(1) $$SI_{1}=1-\left(\frac{SS_{1}}{SC_{1}}\right)$$

A value of 0 would indicate no surround suppression (surround and control matching contrast equal), 1 complete suppression (zero perceived matching contrast). The magnitude of second-order surround suppression was quantified by an index *SI₂* calculated in the same way as for first-order suppression. For the statistical analyses below, first-order and second-order suppression indices were normalized by transforming to z-scores and averaged within subjects to yield a single measure of surround suppression strength for each participant.

## 3 Results

**Fig. 2:**
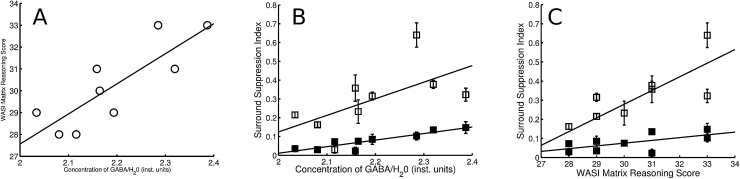
A. Cortical GABA concentration predicts visual intelligence. Correlation between the concentration of GABA/H_2_0 (inst. units) and performance in the Matrix Reasoning IQ task. Line indicates least-squares regression fit. B. GABA concentration predicts strength of perceptual surround suppression. Correlation between the concentration of GABA/H20 (inst. units) and first-order suppression index SI₁ (open symbols) and second-order suppression index SI₂ (filled symbols). Lines indicates least-squares regression fit. Mean suppression indices averaged across eccentricities of 0°, 3°, 6° and 9°. Error bars, standard error of the mean. C. Visual intelligence is correlated with perceptual surround suppression. Correlation between the Matrix Reasoning IQ task scores and SI₁ (open symbols) and SI₂ (filled symbols). Mean suppression indices averaged across eccentricities of 0°, 3°, 6° and 9°. Error bars, standard error of the mean.

We found a strong and significant positive correlation between visual intelligence and cortical GABA concentration (r = 0.83, p = 0.0054), such that subjects with high levels of GABA in primary visual cortex performed better on the matrix reasoning IQ sub-test (Fig. 2A). Higher GABA concentrations were also associated with stronger surround suppression (Fig. 2B); this association was highly significant (correlation between GABA and average normalized suppression indices r = 0.88, p = 0.0017). Moreover, consistent with previous findings [7], high levels of surround suppression were also associated with high visual IQ scores (r = 0.87, p = 0.0021) (Fig. 2C). Both first- and second-order surround suppression were positively correlated with GABA and intelligence (Fig. 2B and C). However, the two measures of surround suppression were not significantly correlated (P > 0.1).

## 4 Discussion

These results demonstrate an association between cortical GABA levels and measures of intelligence, and provide evidence of a relationship between GABA and surround suppression magnitude in typical individuals. A link between GABA and first-order surround suppression has previously been shown for schizophrenic patients, and typical individuals to a lesser extent [9]; our data confirm and extend these findings. Importantly, the finding that GABA levels were also correlated with second-order surround suppression indicates that GABA-mediated inhibition is involved in both forms of surround suppression, and suggests a central role for neural inhibitory mechanisms driving perceptual surround suppression in general [23]. It has been argued that surround suppression mechanisms for motion and contrast involve different neuronal mechanisms, based on the lack of within-subject correlations of these measures [26]. Our results demonstrate that such differences need not preclude the involvement of a common GABA-driven inhibitory mechanism in both forms of surround suppression. Like Yazdani et al. [26], we found little evidence of correlations between first- and second-order surround suppression strength, yet both were correlated with GABA, implying that the differences between the two types of suppression are likely related to other aspects of the neural processing of first- and second-order stimuli.

The observed correlation between GABA and surround suppression is consistent with the notion that visual surround suppression is mediated by inhibitory synaptic connections in the visual cortex [27]. However, it does not in itself provide evidence for a direct causal link or reveal the underlying mechanism. The results of physiological studies investigating this issue have been somewhat mixed; for example, Adesnik et al. [28] reported evidence that local intracortical GABAergic inhibition mediates suppression in V1, whereas Liu and Pack [16] found that directly manipulating GABA levels in area MT had no effect on surround suppression. Ozeki et al. [29] reported that surround suppression in V1 results in a reduction rather than increase in inhibition, which would seemingly argue against a direct role for inhibition underlying surround suppression. However, they showed that this result can be accounted for by a cortical circuit model in which surround suppression is mediated by an inhibition-stabilized network (ISN). In this model, the role of inhibition is to stabilize excitatory responses to allow graded responses to stimuli, and surround suppression arises through a reduction in excitation rather than by an increase in inhibition. The strength of surround suppression is influenced by the strength of inhibitory input to individual neurons (consistent with the correlation between GABA and surround suppression in our results and those of Yoon et al. [9]), but because global effects of surround suppression are associated with the overall balance between excitation and inhibition, local manipulations of inhibition (e.g. by application of bicuculline or GABA) should have little effect on surround suppression, which may explain the results of Liu and Pack [16].

Combined with our finding of a strong positive correlation between surround suppression strength and intelligence, and between intelligence and GABA, these results provide a plausible candidate neural substrate for Melnick et al.’s [7] proposal that the link between surround suppression and IQ is the suppression of irrelevant information. Our findings are also consistent with indirect evidence for a link between GABA and the exclusion of irrelevant information [30]. These findings may be interpreted within the framework of the ISN model of Ozeki et al. [29], which proposes that the primary role of GABA is to stabilize network activity, by considering irrelevant information as a form of destabilizing input. According to this interpretation, GABAergic inhibition may be thought of as effectively serving to increase the cognitive or perceptual signal-to-noise ratio. Such an interpretation may also provide an explanation for the finding by Sumner et al. [14] that inter-individual variations in frontal cortical GABA levels are predictive of the speed of motor decisions.

Although our results provide evidence for a link between GABAergic neural inhibition and visual intelligence, the specific mechanisms and nature of this link remain to be elucidated. The generalisability of our findings is necessarily limited by the relatively small sample size, as is common with much of the MRS literature [13] and the study focused solely on the visual domain. The IQ subtest relies heavily on visual reasoning, only low-level visual abilities were considered and GABA measurements were taken from the visual cortex. Individual variations in GABA levels need not be uniform across different cortical areas [31], suggesting any relationship between GABA and cognitive performance may be domain-specific. However, IQ in non-sensory domains has also been linked to visual surround suppression [7] and low-level perceptual tasks in non-visual domains have been linked to IQ [3], [4]. Moreover, differences in GABA levels in frontal cortex are associated with non-visual task performance [14]. Further investigation will be necessary to determine whether similar links between GABA and measures of intelligence are also present in other sensory domains and in non-sensory cortical systems, and whether suppressive neural processes in these systems also involve GABA-mediated neural inhibition.
